# Marine heatwaves shift intertidal marine communities in the SW Atlantic

**DOI:** 10.7717/peerj.20858

**Published:** 2026-02-25

**Authors:** Ana Carolina Azevedo Mazzuco, Daniela Y. Gaurisas, André Vassoler, Gabriel C. Coppo, Carla Frecchiami de Oliveira Pacheco, Gustavo Alves Alcure Araujo, Fernanda Maria Menezes Alves, Angelo F. Bernardino

**Affiliations:** 1 UNESCO-IOC, Oostende, Belgium; 2 Department of Oceanography, Universidade Federal do Espírito Santo, Vitoria, Espirito Santo, Brazil

**Keywords:** Marine heatwaves, Benthos, Intertidal, Atlantic, Macroalgae, Climate change

## Abstract

**Background:**

Accelerating rates of ocean warming are a global threat to coastal marine ecosystems. More frequent heat extremes, also known as marine heatwaves, are likely to cause severe impacts on marine ecosystems as species may be unable to tolerate, adapt to, or recover from these events.

**Methods:**

In this study, we examined the association between the occurrence of marine heatwaves and benthic cover at intertidal reefs in the SW Atlantic. To investigate these effects, we monitored *in situ* temperatures. We obtained remote-sensing satellite measurements of essential ocean and biodiversity variables at a long-term marine observatory (LTER) on the SE coast of Brazil. The dataset resulted in monthly (December 2017 to May 2022) monitoring of intertidal macroalgal beds and coastal meteo-oceanographic conditions.

**Results:**

Our results revealed that temporal changes in macroalgal cover during the study period were highly correlated (>80% association) with marine heatwaves, which were the dominant extreme weather conditions in this region. Stronger (intensity effect) and prolonged (weeks) events result in a significant decrease (38% loss) in macroalgal cover with impacts on benthic richness and diversity both locally and regionally. The impacts were more pronounced and drove succession on dominant macroalgal taxa (brown and red algae), which were the least resilient to high temperatures. Although there were indications of macroalgal recovery after 2020, the community did not return to the pre-heatwave composition, revealing that assemblage succession over these macroalgal beds may occur at multi-year time scales. Our study supports previous research indicating that marine heatwaves are becoming more frequent along the Brazilian coast. Given the prolonged period for assemblages’ recovery, we can expect marked decreases in coastal biodiversity in the SW Atlantic.

## Introduction

The effects of global warming on marine ecosystems are widespread, significant, and persistent throughout this century, leading to the loss of marine biodiversity over large scales (Bindoff et al., 2019; Collins et al., 2019). Coastal ecosystems such as coral reefs and rocky shores are particularly sensitive to climate change impacts due to their vulnerability to multiple stressors and exposure to the most anomalous environmental conditions (Poloczanska et al., 2016; Kunze, Wölfelschneider & Rölfer, 2021). In recent years, key intertidal species have disappeared across extensive areas, while many others have declined in abundance or been replaced by more resilient taxa (Poloczanska et al., 2016). At many sites, overall biological abundance and biomass have already been significantly reduced in response to warming, and entire coastal ecosystems are undergoing substantial changes (Pontavice et al., 2020; Gorman et al., 2020; Pereira et al., 2022). Considering the projected 1.5 °C global warming by 2050, monitoring marine assemblage succession and recovery is a crucial step toward assessing climate impacts (IPCC, 2022a, IPCC, 2022b), and essential to guiding conservation actions and preparing for future biodiversity loss (Muelbert et al., 2019; Frazão Santos et al., 2020).

Extreme weather events are rare and intense environmental phenomena, typically defined by their frequency of occurrence in a given location (Hobday et al., 2016; Schlegel et al., 2017). Among these, marine heat waves (MHWs) are localized periods of anomalously elevated sea temperatures that can persist for months and cover vast oceanic areas (Hobday et al., 2016). Over the past two decades, their global frequency has roughly doubled, and they have become increasingly prolonged, intense, and widespread (Collins et al., 2019). Therefore, determining community variability in coastal ecosystems under rapid environmental change is challenging, requiring multivariate approaches to disentangle the numerous processes driving biodiversity shifts (Borja et al., 2020).

A major premise is that extremely high sea temperatures limit the growth and survival of most marine species and are expected to be associated with ecological changes where warming is more intense (Luypaert et al., 2020). However, in nearshore ecosystems, exposure to air, wave impact, and radiation during low tides are additional factors that affect the structure and functioning of coastal benthic assemblages (Kunze, Wölfelschneider & Rölfer, 2021). In these habitats, marine heatwaves can be extremely harmful, exceeding the thermal tolerance thresholds of coastal species and causing prolonged ecological shifts (Poloczanska et al., 2016; Smale et al., 2019). These events can lead to the decline of coastal foundation species (*e.g.*, reef-building algae and corals; Thomson et al., 2015; Smith et al., 2024), potential disruptions on ecosystem functioning (De Luzinais et al., 2024), and, in extreme cases, trigger mass mortality events (MMEs) worldwide (Short et al., 2015; Garrabou et al., 2022; Pereira et al., 2022). The intensity, duration, and frequency of these simultaneous or alternating conditions are expected to drive large-scale changes in coastal assemblages and aggravate future climate impacts (IPCC, 2022b).

The response of intertidal marine assemblages to marine heatwaves have been evaluated across several regions, including the northeastern Atlantic (Mieszkowska et al., 2019), northwestern Pacific (Ishida et al., 2023), northeastern Pacific (Sanford et al., 2019; Weitzman et al., 2021) and the SW Atlantic (Nauer et al., 2022). These responses vary widely, ranging from negative impacts (*e.g.*, mortality of habitat-forming species and loss of biodiversity), to neutral or resilient outcomes, and even positive responses such as increased abundance and expansion of warm-affinity taxa (Smale, Wernberg & Vanderklift, 2017; Sanford et al., 2019; Nauer et al., 2022; Ishida et al., 2023). However, there remains limited information on how these events and synchronous oceanographic conditions influence assemblage succession along interannual time scales for many coastal regions, especially for the South Atlantic, contributing to the underestimation of global climate impacts.

Ocean observing systems and marine long-term ecological programs globally provide continuous information on climate and oceanic conditions at multiple scales (Muelbert et al., 2019; Tanhua et al., 2019). This data, openly available in online repositories, are highly valuable for biodiversity assessments, especially in climate change research (Canonico et al., 2019). High-resolution ocean-climate information from the last 100 years can now be obtained freely, enabling temporal comparisons at scales relevant to marine ecological processes (*e.g.*, ERDDAP; Wilson, Robinson & Simons, 2020). The multivariate availability of oceanographic parameters offers an advantage when investigating potential drivers of marine biodiversity change (Canonico et al., 2019), allowing the evaluation of co-variability and the ranking of environmental influences on species assemblages or community, prerequisites for modeling and predicting biodiversity patterns. Even for coastal habitats where stressors multiplicity is high, coordinated *in situ* and satellite remote-sensing monitoring makes it feasible to estimate warming effects at regional and local scales (Goodell, Stamoulis & Friedlander, 2018; Mazzuco et al., 2019; Mazzuco & Bernardino, 2022). The outcomes of these studies support conservation and management efforts worldwide, contributing to ocean sustainability (Evans et al., 2019).

Along the Southwestern Atlantic coast, marine ecosystems are highly vulnerable to climate change impacts, including droughts and short-term extreme high temperature events (Rodrigues et al., 2019; Brauko et al., 2020; Gomes & Bernardino, 2020; Pereira et al., 2022). Intertidal and coastal marine ecosystems have been experiencing loss of biodiversity and biomass due to recent warming (Duarte et al., 2020; Anderson et al., 2021). Although warming effects may be more evident in coral-dominated sites than in macroalgae-dominated habitats, recent reviews report significant reduction in several macroalgal species across the region, related to warming intensification (Mazzuco, Stelzer & Bernardino, 2020). Nevertheless, major gaps remain regarding the environmental drivers of these biodiversity changes along the Brazilian coast, including assemblage succession after events and recovery patterns. While there is strong evidence of negative impacts of high temperatures on these coastal habitats, there is limited information on whether these ecosystems can recover from multiple extreme events of varying intensity.

To assess the impacts of climate change on SW Atlantic coastal ecosystems, we monitored oceanographic conditions for over three years (2018 to 2022) at a Long-Term Ecological Research (LTER) site in the Eastern Marine Ecoregion of Brazil and identified the occurrence and duration of marine heatwaves. Concurrently, we monitored *in situ* patterns of benthic assemblage succession at intertidal macroalgal beds. Our study aimed to identify shifts in essential biodiversity variables and ocean-climate indicators to test the hypothesis that intertidal macroalgal beds are highly impacted by marine heatwaves, but exhibit taxa-specific responses that reflect different susceptibility to heatwaves and recovery times. We also described marine heatwaves occurrence, persistence, and intensity in the studied region. This study advances the understanding of climate change effects on macroalgae-dominated habitats and provides valuable insights into ecosystem resilience and ecological succession under extreme climatic conditions for the Eastern Marine Ecoregion of Brazil.

## Materials & Methods

### Study area and field surveys

**Figure 1:**
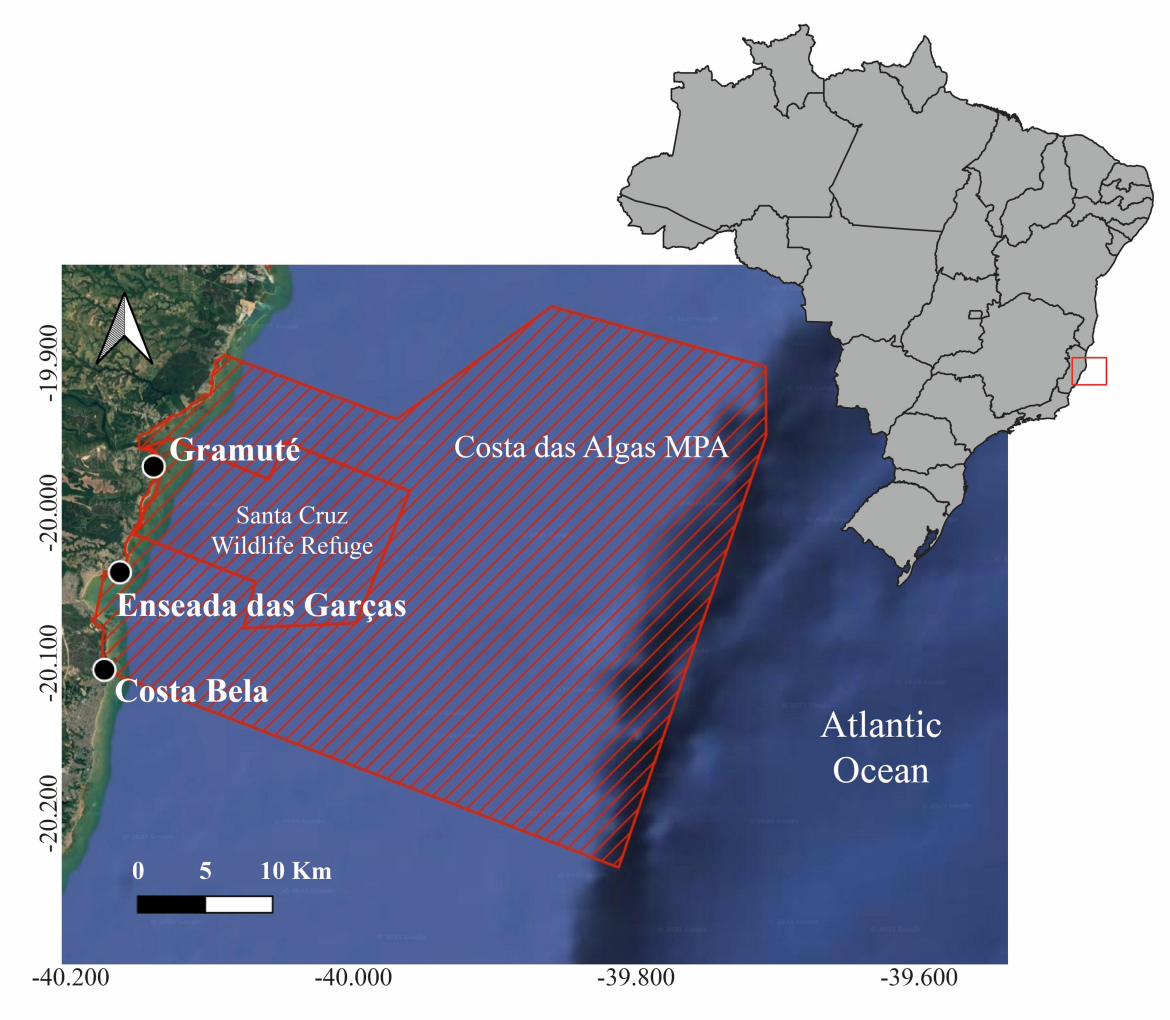
Location of sampling sites. Location of the LTER HCES sites (black symbol) inside the Costa das Algas Marine Protection Area and Santa Cruz Wildlife Refuge (red line), located on the Eastern coast of Brazil. Map component sources: Esri, NASA, NOAA, USGS, Garmin, Foursquare.

This study was conducted in a marine protected area (MPA) within the Eastern Brazil Marine Ecoregion (Área de Proteção Ambiental Costa das Algas; environmental permit granted by Instituto Chico Mendes #57819-9; Fig. 1). It is a tropical region, with an annual average air temperature of 25 °C, which has experienced significant warming trends with frequent and intense positive temperature anomalies (up to +1.5 °C above decadal means) during the last decade (Bernardino et al., 2015). The coastal zone is characterized by sandy beaches and intertidal lateritic reefs with extensive macroalgal beds, under oceanographic conditions typically influenced by E-NE winds from the South Atlantic high-pressure system and E-SE wave swells (Mazzuco et al., 2019; Mazzuco, Stelzer & Bernardino, 2020). Episodic upwelling of the South Atlantic Central Water mass (SACW), a deep, cold, nutrient-rich water mass that enhances primary productivity, mainly occurs during spring and summer (Quintana et al., 2015). Coastal seascapes in the region are dominated by the ‘Warm, Blooms, High nutrients’, and ‘Tropical Seas’ classes (<70% of coastal areas; (Mazzuco & Bernardino, 2022; Coppo et al., 2024).

Field campaigns were conducted monthly to trimonthly (with gaps due to COVID-19 lockdown period or logistical gaps) from December 2017 to May 2022 at a reef area monitored by the LTER Coastal Habitats of Espírito Santo PELD-HCES (Gramuté beach, Lat. 19°57′58.68″S, Long. 40°8′2.4″W; Fig. 1, Table S1). Reef benthic biodiversity was estimated along five replicated 20-m length photo-transects using a GoPro camera. Images were obtained during low-tide by photographing an area within a 0.25 m^2^ quadrat, taking one photo every two m along the transect, for a total of 10 photos per transect (50 photos per month). Transects were positioned parallel to the reef fringe approximately five meters apart. Images were processed in Coral Point Count with Excel Extensions Software (CPCe), where benthic taxa were visually identified under 20 random points within a 100-point grid (Carleton & Done, 1995; Mazzuco, Stelzer & Bernardino, 2020). Image processing generated benthic primary cover datasets with taxonomic resolution up to the ’genus’ level. Quality control involved multiple observers (*n* = 5) cross-validating image subsets, as well as *in situ* sampling and validation.

To evaluate the spatial extent and persistence of regional changes in these macroalgal beds, three additional surveys were conducted at two other sites within the MPA (Enseada das Garças beach, Lat. 20°2′12.84″S, Long. 40°9′50.4″W; and Costa Bela beach, Lat. 20°6′20.52″S, Long. 40°7′40.8″W) and the LTER site (Gramuté). These surveys followed the MBON Pole to Pole of the Americas Project rocky shore *in situ* monitoring protocol (MBON Pole to Pole, 2019), which consists of visually identifying the primary cover (100-point grid) and the presence or absence of organisms in a 0.25 m^2^ quadrat along different reef strata (high tide, mid-tide, and low tide) with a total of 30 replicates per survey. These surveys were carried out in the summers of 2018, 2019, and 2020 (data for the following years are not available). Only data from the low-intertidal MBON Pole to Pole samplings were included in this analysis, which was comparable to the LTER samplings (10 replicates per site per sampling campaign).

**Table 1:** Meteo-oceanographic variables monitored and respective data sources and resolution.

Climatic indicator	Monitores variables	Abbreviation	Sources	Spatial resolution
Surface temperature	Air Temperature at the surface level	AirT mean	NCEP/NCAR Reanalysis 1 (Kalnay et al., 1996)	0.5° lat/long
		AirT anomaly	NOAA Merged Land Ocean Global Surface Temperature Analysis (Vose et al., 2012; Huang et al., 2017)	5° lat/long
Ocean heat	Sea surface temperature	SST mean	Multi-scale Ultra-high Resolution (MUR) SST Analysis (NOAA, 2020)	0.01° lat/long
		SST anomaly		
Sea level	Significant Wave Height	SWH mean	Near Real Time WAVE-TAC multi-mission and Aviso altimeter data	2° lat/long
	Significant Sea Height	SSH anomaly	Sea Surface Height Anomalies from Altimetry (Leuliette & Scharroo, 2010)	0.25° lat/long
Storm surge	Precipitation	Precip mean and anomaly	CHIRPS Version 2.0 UCSB Climate Hazards Group (Funk et al., 2014; Funk et al., 2015)	0.05° lat/long

### Meteo-oceanographic monitoring

Climatic and marine conditions were characterized using key indicators and essential meteo-oceanographic variables (surface temperature, ocean heat, sea level, wave impact, and storm surge; Table 1). Data were obtained from satellite remote sensing observations with data freely available from the National Oceanic and Atmospheric Administration (NOAA) CoastWatch/OceanWatch Program and the Copernicus Marine Service. Monthly gridded data was retrieved from the online repositories and averaged for the study period. To ensure consistency across environmental datasets, all variables were resampled to a common spatial resolution using the nearest area possible (site scale, Latitude 19°57′58.68″S; Longitude 40°8′2.4″W). Each sampling site was represented by the centered pixel closest to its geographic coordinates. We also acknowledge the variation in pixel size among the original satellite datasets, which may influence coastal representation and is considered in the interpretation of the results. However, these differences are not expected to significantly affect the spatial patterns observed in this study. For each variable, both mean and anomalous conditions were evaluated (Table 1). Marine heatwaves were characterized in terms of their frequency, duration, and intensity, and analyzed in relation to tidal variability (low-tide and high-wave conditions). Accordingly, MHWs were considered as prolonged discrete anomalous events that lasted for five or more days, with temperatures warmer than the 90th percentile based on a 30-year historical baseline period (Hobday et al., 2016; Hobday et al., 2018). Therefore, our baseline corresponds to the three decades immediately preceding the sampling years, ensuring that the climatology reflects the local long-term conditions relevant to our study period.

Complementarily, we evaluated extreme conditions at the local scale, adding air exposure (low-tide) and storm surge (wave height) to the assessment. Low-tide, high-temperature, and high-wave events were identified by comparing the variances of daily meteo-oceanographic conditions at the study site with those of historical regimes (*e.g.*, (Amorim et al., 2012). Temperature in-situ data was collected using a HOBO MX TidbiT 400 (MX2203) temperature logger, deployed at 0.3 m depth at the study site. The logger recorded temperature at 1-hour intervals, and data were visually inspected and screened for anomalous values before analysis. We also compared the in-situ data with satellite-derived SST for the same dates and site. The two datasets showed a strong positive correlation (*r* = 0.917; *p* < 0.001; Fig. S1), confirming the consistency of the logger measurements. Low-tide high-temperature events were considered when diurnal low tides exposed the macroalgal beds for longer periods (≥4 h), which is equivalent to a minimum tide height ≤0.3 m, and co-occurred with high positive daily sea temperatures (≥0.5 °C above the seasonal mean; *e.g.*, McPhaden, Zebiak & Glantz, 2006). High-wave events were considered when waves exceeded a period of 10 s and a height of ≥ 1.5 m (Pianca, Mazzini & Siegle, 2010). When more than one event was detected, the events were considered separate by a period of 5 days according to the typical meteorological cycles in the region (cold front passages; (Pianca, Mazzini & Siegle, 2010). Event duration (total number of days) and intensity (sea surface temperature anomaly) were also characterized for each month. These compound events were flagged in the dataset and subsequently considered in the correlation analysis with benthic assemblage data.

### Data analysis

Temporal (yearly and seasonal) variability in climatic and marine indicators (surface temperature, ocean heat, and sea surface level) and MHWs were evaluated through two-way Analyses of Variance (ANOVA; (Underwood, 1997). The dataset comprised of 32 months, divided into three replicates per season, four seasons within a year (summer, fall, winter, and spring), and five years (Dec-2017 to Nov-2018; Dec-2018 to Nov-2019; Dec-2019 to Nov-2020; Dec-2020 to Nov-2021; Dec-2021 to Jul-2022). Data were transformed (log x + 1) when needed to fit the assumptions of ANOVA (normality and homogeneity of variances) after verification by Kolmogorov–Smirnov and Cochran tests (Cochran, 1941; Marsaglia, Tsang & Wang, 2003). Significant ANOVA results were followed by *post hoc* pairwise Tukey HSD tests. Differences between extreme and regular conditions were evaluated with canonical discriminant function analyses (DFA) that tested categories based on the numerical relationships between the occurrence, duration, and impact of the events. DFA results were interpreted based on the linear discriminant coefficients, and jackknife re-samplings were included in the analyses to test the accuracy of the classifications by DFA (Tukey, 1949).

Temporal trends in macroalgal beds (% cover, richness, and diversity) were analyzed through generalized additive models (GAM; Hastie & Tibshirani, 1990; Hastie, 1991), built using the average values per transect for each month and the accumulated sampling days. Percentage cover was calculated independently for each taxonomic group (corals, macroalgae, and other epibenthos), randomly choosing different quadrats (3 per group) within a transect. Richness (the number of taxa) and diversity (Shannon–Wiener index) were evaluated using the average of all quadrats available for a transect (9–10). GAM results included parametric and non-parametric parameters (F and p) and were complemented by unbalanced ANOVAs and pairwise Tukey HSD tests to evaluate variability between years, seasons, and months.

The temporal variability within each taxonomic group (Rhodophyta, Chlorophyta, Phaeophyta, Anthozoa, and Other) was evaluated by graphical patterns and canonical analysis of principal coordinates (CAP). We compared the composition of macroalgal beds to extreme event conditions using a CAP (Anderson & Willis, 2003). This analysis provided ranked information on community vulnerability to climate change, highlighting conditions most closely related to the shift in assemblage composition and abundance. We assumed *α* = 0.05 for all analyses and determined the significant *p*-values using the Benjamin-Hochberg false discovery rate (FDR) method for multiple post hoc comparisons (Verhoeven, Simonsen & McIntyre, 2005). Graphical and analytical processing were performed in Panoply 4.12.11 (Schmunk, 2023) for remote sensing data extraction, Numbers (Apple Inc.) for charts, and R project (R Core Team, 2023) for statistics (R packages ‘stats’ for general calculations, ‘GAD’ (Sandrini-Neto & Camargo, 2011) and ‘outliers’ (Komsta, 2011) for ANOVAs and posthoc tests, ‘vegan’ (Oksanen et al., 2013) for PERMANOVA and CAP, ‘’rich’ (Rossi, 2011) for the calculation of ecological indexes, and ‘gam’ (Hastie, 2019) for modeling.

## Results

### Meteo-oceanographic conditions

**Figure 2:**
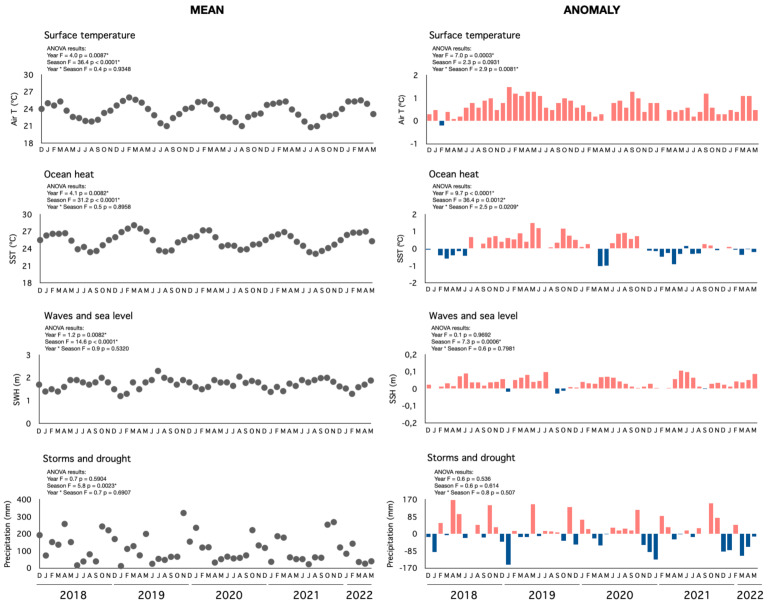
Mean and anomalous regional climatic indicators. Monthly variation of the mean and anomalous regional climatic indicators (surface temperature, ocean heat, sea level, storms, and drought) during the study. Note: air temperature: Air T, sea surface temperature: SST, significant wave height: SWH, and precipitation: Precip.

There were marked changes in the marine and meteorological conditions during the study, where air and sea temperatures showed the highest variability (Fig. 2, Table S2). Overall, monthly temperatures were warmer than the historical averages, ranging from 20.8 to 26.0 °C in the air (average 23.5 °C) and from 23.1 to 28.1 °C in the sea (Fig. 2). Sea temperature anomalies were positive during 51 out of 54 monitoring months (≥1 °C for 13 months) and during 23 months (≥1 °C for 3 months) for air measurements. Temperatures were the highest in 2019, with maximum anomalies in May, June, and October (ANOVA *F* = 4, *p* < 0.01; Fig. 2). Larger seasonal differences in air and sea temperature were registered between the summer and winter months (Table S2). Sea surface height and precipitation showed only seasonal variations. Wave heights ranged between 1.2 and 2.5 m (average 1.7 m) and sea level anomalies between −0.02 and 0.11 m (average 0.03 m), with higher measurements during winter (ANOVA *F* = 1.2, *p* = 0.008; Fig. 2; Table S2). Mean rainfall was low during most of the monitoring (averaging 11.8 to 321.5 mm), with the highest volumes in the spring and summer (Fig. 2; Table S2).

**Figure 3:**
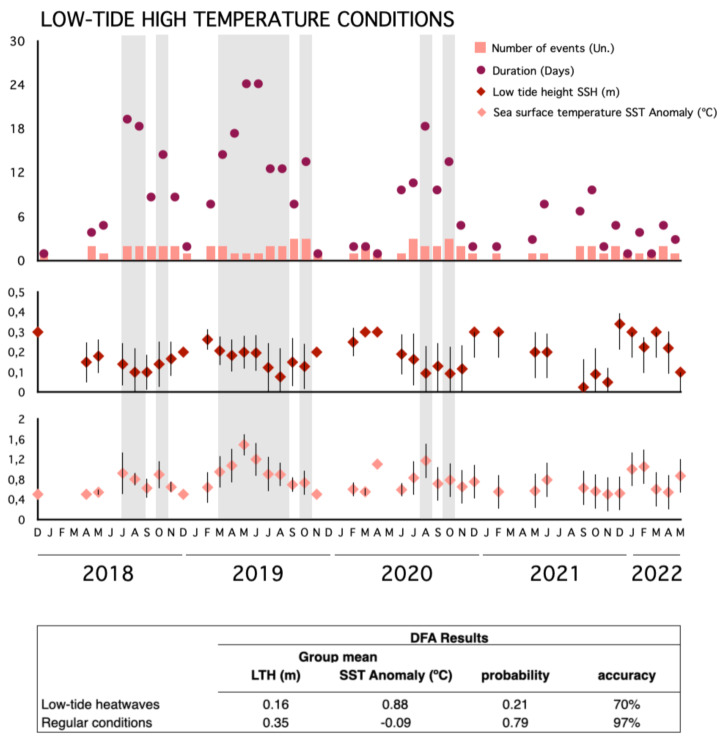
Occurrence of marine heatwaves during the study period. Monthly assessment of extreme conditions (low-tide high temperature, high-wave) from December 2017 to May 2022 at the study site. Upper panel: Bars indicate number of events, circles indicate event duration. Middle panel: Low tide height (m). Lower panel: SST anomaly in Celsius.

According to the climatological time series, 22 marine heatwave events were registered at the study site between December 2017 and May 2022 (Table S3). Five of them sustained anomalous warmer conditions for more than 15 consecutive days, and one of them lasted for 47 days between April and June 2019, with a maximum sea temperature anomaly of more than 4 °C. Daily extreme warm meteo-oceanographic conditions with simultaneous low-tide heatwaves were detected in the region (Fig. 3). At least two low-tide high temperatures were registered almost every month with a duration of up to 25 days (observed in May and June 2019), but with a lower probability of occurrence (21%) compared to the regular conditions (79%; Fig. 3). Average low-tide height and SST anomaly during these events were 0.17 m and 0.62 °C, respectively. Longer and more persistent low-tide high temperatures were observed from June to October 2018, March to October 2019, and June to October 2020 (Fig. 3, Table S4). Winter and spring were the seasons with the most low-tide, high-temperature days, with averages of 8 and 12 days per month, while summer had the lowest average (2 days per month; Table S4). High-wave rainstorms were far less frequent than heatwaves and did not vary between years (<1% probability of occurrence; Fig. 3). During these rare events, the average wave height was 1.8 m and precipitation was 17.5 mm, with low variability between monitored years (Fig. 3; Table S4).

### Temporal variability of macroalgal beds

**Figure 4:**
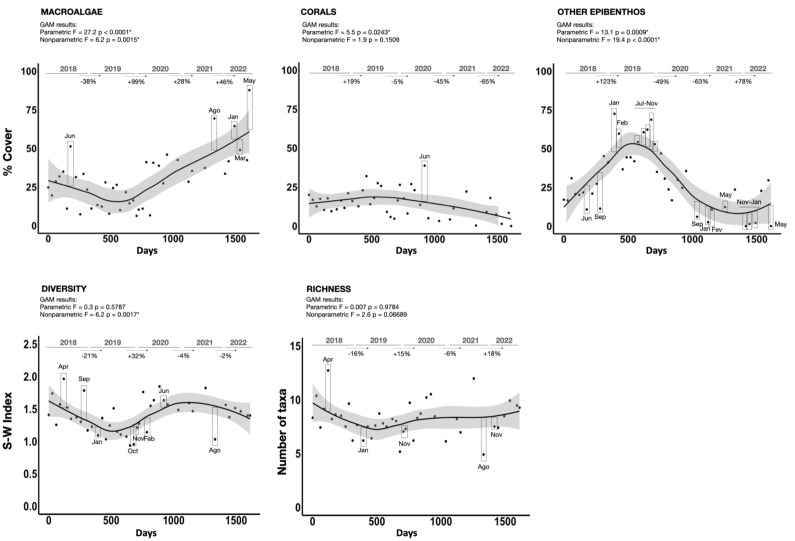
Temporal trends of intertidal benthic assemblage composition. Temporal trends of intertidal benthic cover (macroalgae, corals, and other epibenthos), richness, and diversity from December 2017 to May 2022 described by generalized additive model adjustment. Major increases (+) and decreases (-) in average measurements between years are described on top of each chart. Significant monthly contrasts according to ANOVA results are highlighted by line boxes.

**Figure 5:**
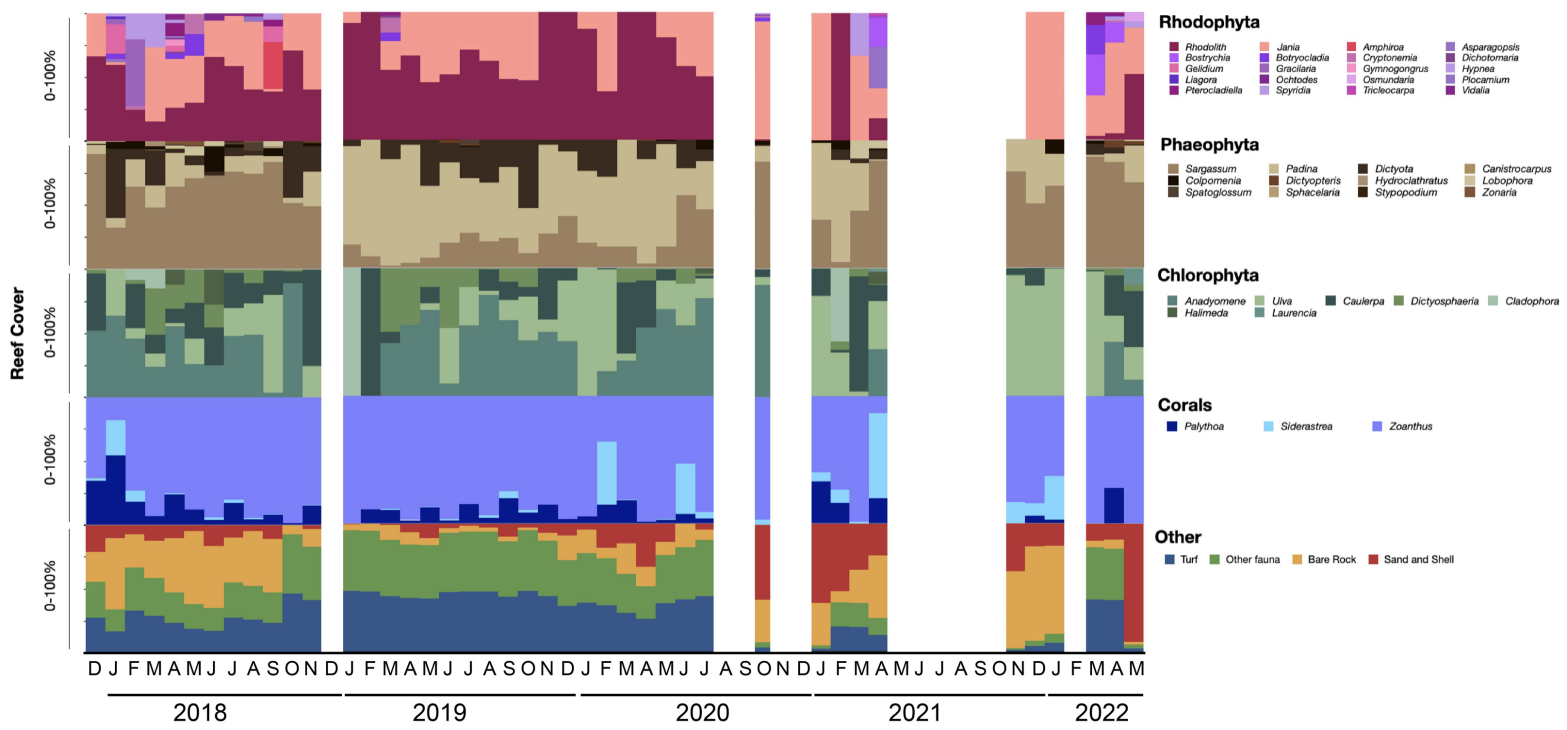
Benthic assemblage composition (% cover). Variation in benthic assemblage composition (% cover) within dominant taxonomic groups. Blank periods indicate data gaps.

Over 53 taxa were identified at the monitored sites, mostly identified at the genus level, with a monthly average diversity of 1.4 and a richness of eight taxa (Fig. 4; Table S5). Intertidal areas were covered by macroalgal beds, which accounted for an average of 30% of the benthic surface and 38 genera. Among these macroalgae, rhodoliths (composed of several calcareous species) and common Corallinaceae (*Corallina* and *Jania*, undistinguishable through our image resolution) were the most abundant. Rhodophyta, *Sargassum* and *Padina*, Phaeophyta, and Chlorophyta alternated dominance among four genera (*Anadyomene*, *Ulva*, *Caulerpa*, and *Dictyosphaeria*; Fig. 5). Three corals (*Palythoa*, *Siderastrea*, and *Zoanthus*) covered an average of 15% of the intertidal zone. The surrounding living area (29%) was covered by diverse epibenthic species, including mobile invertebrates (gastropods, echinoderms, and hermit crabs), encrusting organisms (sponges and anemones), and turf (thick biofilm and thin filamentous algae; Table S5; Table S6; Table S7).

There was high variability in the benthic % cover and assemblage composition at the three temporal scales, but most differences were observed inter-annually (Figs. 4 and 5; Table S8). The major change was registered in 2018-2019 when there was a 38% decrease in total macroalgae cover, followed by a 19% increase in corals and a 123% increase in other epibenthic species (ANOVA *F* = 27.2, *p* = 0.0001; Fig. 4; Table S8; Table S9; Fig. S2). Following the summer of 2018, we observed a notable decline in the cover of red algae species with a switch of dominance among the brown algae from *Sargassum* to *Padina*, and an increase in *Anadyomene* within the green algae (Fig. 5). Those changes were followed by a bloom of turf and other fauna (>50%) and a simultaneous decrease in bare rock. Macroalgae gradually recovered in the following years, reaching their maximum abundance in 2022, while corals and other epibenthic organisms decreased to a minimum (Fig. 4). *Zoanthus* remained the most abundant coral during the study, with a slight decrease in the dominance for *Palythoa* and *Siderastrea* in 2020–2021 (Fig. 5). Diversity in the macroalgal beds reached maximum values in 2018 and the lowest values at the end of 2019 and again in 2021 (ANOVA *F* = 6.2, *p* = 0.001; Fig. 4; Fig. S2).

We also observed seasonal and monthly differences in these benthic assemblages (Table S6, Table S7; Figs. 4 and 5). Macroalgae were more abundant (>50%) in June 2018, August 2021, and January and May 2022. The largest coral cover was registered in June 2020 (>30%). Other epibenthos showed the highest variability between months, primarily due to differences in the abundance of turf (Fig. 4). There was more heterogeneity in the assemblage composition during specific seasons, with spring exhibiting greater diversity, and winter showing higher richness (Table S6). April and September 2018 were the months with the highest benthic diversity and richness, and in August 2021, these parameters reached minimum levels (Tables S6 and S7).

### Multivariate analysis

**Figure 6:**
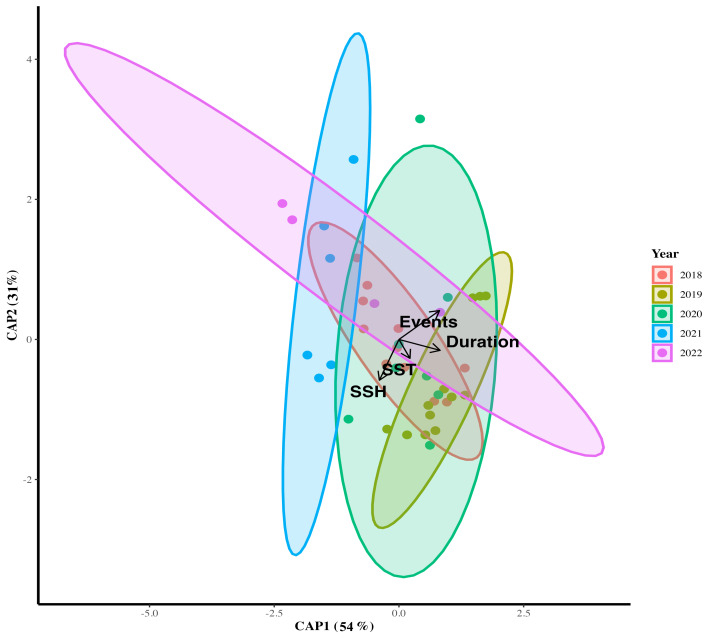
CAP ordination of benthic assemblages along MHV events. Canonical analyses of principal coordinates (CAP) ordination of benthic asssemblages during periods of MHV. Vectors are based on Spearman correlation values > 0.5 for MHV events, duration, SST, and SSH. The proportion of data explained by axis 1 and 2 are given. Color indicate year groups (red for 2018, yellow for 2019, green for 2020, dark blue for 2021, and pink for 2022).

The CAP revealed a distinct shift in the composition of the macroalgal bed assemblage across years (*F* = 1.654; *p* = 0.027; *df* = 4; Table S10), which aligned strongly with the vectors corresponding to MHW metrics (Fig. 6). Both CAP axes explained 84% of the data distribution, suggesting a significant effect of cumulative event occurrence, duration and ocean status represented by temperature and SSH. The number and duration of events was mostly relevant (54%) explaining the ordination of benthic assemblages across years. The years of 2020, 2021 and 2021 were more dissimilar across the ordination space, with the year 2021 clustered far apart the previous period likely reflecting stronger compositional dissimilarity across time. The period of 2020–2022 was correlated with weaker or shorter MHW events and, probably, suggesting a recovery process of the macroalgal bed assemblage (Fig. 6).

## Discussion

Our study shows that extreme weather events such as marine heatwaves drive substantial ecological changes in the diversity, richness, and structure of coastal macroalgal beds in the Tropical Southwestern Atlantic. These findings reinforce and expand upon the growing global evidence that the increasing frequency and duration of marine heatwaves are affecting benthic communities worldwide (Oliver et al., 2018; Gorman et al., 2020). Specifically, we demonstrate that the co-occurrence of marine heatwaves and low-tide exposure conditions impact shallow intertidal habitats in the Eastern Marine Ecoregion of Brazil, resulting in marked changes in the macroalgal assemblages. Our results support that these ecosystems are frequently exposed to extremely high temperatures and are likely to be among the most severely impacted by climate change and anthropogenic activities (Rivetti et al., 2014; Martinez & Altvater, 2024).

In recent years, the effects of elevated temperatures on benthic macroalgal forests have received increasing global attention; research has shown that warming has directly affected the abundance, distribution, and geographic range of many macroalgal species. However, most research has focused on kelp-dominated systems (Assis et al., 2014; Martinez et al., 2018; Smale, 2020). Several studies in the SW Atlantic have documented the impacts of extreme warming on coastal macroalgal communities (Nauer et al., 2022), but with stronger emphasis on coral reef bleaching and mortality (Duarte et al., 2020; Pereira et al., 2022; Da Silva, Kampel & Nakamura, 2025). By coupling a simple, standardized photo-transect monitoring approach with satellite remote sensing data, our study provides a robust and accessible framework that reveals clear changes in the composition and diversity of macroalgal bed communities during marine heatwaves—an approach that strengthens long-term monitoring efforts in tropical coastal ecosystems.

As observed globally, macroalgae-dominated sites are significantly impacted by high temperatures, which, in combination with low tides, may create harsh conditions for the survival of less tolerant species (Gorman et al., 2020). The Eastern Marine Ecoregion of Brazil has one of the highest diversities of macroalgae in the world, with seaweed and coralline beds found across all coastal habitats from estuaries to mesophotic areas of the continental shelf (Mazzuco et al., 2019; Sissini et al., 2022 Tuya et al., 2023). These submerged marine forests support a rich community of invertebrates and fish, providing numerous services to humans on the coast that are still underestimated for SW Atlantic habitats (Mazzuco et al., 2019; Schubert et al., 2025). The declines in macroalgae cover recorded in our study were more intense in years with anomalous temperature increases, mirroring observations from other sites in Brazil and globally (Straub et al., 2019; Smale, 2020; Nauer et al., 2022), but in discordance with findings in the NW Pacific, where there was an increase in macroalgae abundance during and after a MHW (Ishida et al., 2023). However, biological tolerance to heating may lead to an alternate composition of low-diversity macroalgal beds in the long term (Agostini et al., 2021; Nauer et al., 2022). In our study, fucoids (*e.g.*, *Sargassum)* were negatively affected by MHWs, while turf-forming seaweeds (*e.g.*, *Ulva*) were promoted, a finding consistent with those in Chile (Soto, 1985) and Western Australia (Wernberg et al., 2013), which were related to periods of warming anomalies. This may indicate that an increase in turf-dominated reefs in the SE coast of Brazil is likely if marine heatwaves become more frequent.

The impacts of MHW conditions were stronger on brown (*e.g.*, *Sargassum* was replaced by *Padina*) and red algae (a decline from 22 to only 2 taxa), which were replaced by ephemeral species (*e.g.*, turf, zoanthids). Red and brown algal beds are the most common live cover in these reefs, providing habitat, refuge, feeding, and nursery grounds for many marine species (Mazzuco & Bernardino, 2022). They are important components in the carbon cycle and are distributed along extensive coastal stretches in the studied region (Vassoler et al., 2025). Both macroalgal groups do not tolerate prolonged exposure to intense radiation and high temperatures (Short et al., 2015; Nauer et al., 2022), and their decline during MHWs should be expected. During our monitoring, MHW conditions persisted for over 15 days in the study region, with temperature anomalies reaching up to 4 °C above the long-term average and being exposed to air and solar radiation for about 4 h daily, which can be lethal for many intertidal algal taxa.

Although macroalgae were the dominant group and our primary focus, changes in their cover also coincided with shifts in coral and other epibenthic taxa, suggesting broader community restructuring. *Zoanthus* was the dominant coral in the study, while scleractinian coral genera, such as *Palythoa* and *Siderastrea,* were less represented. *Siderastrea* was nearly absent during the 2018–2019 MHW period and reappeared again at the end of 2019 and early 2020. Species of *Siderastrea* have demonstrated high resistance to thermal stress compared with other scleractinian corals, which enables them to recover easily after anomalously warm ocean temperatures (Pinheiro et al., 2017). However, when warming events are intense or persist for long periods—such as during El Niño—they can experience bleaching and become more susceptible to various coral diseases (Ferreira et al., 2023). Our findings suggest that *Siderastrea* was impacted by the MHW in 2019 and showed signs of recovery after that MHW, remaining resilient to subsequent heat waves that were not as intense.

Following these MHWs and low-tide periods, assemblage recovery was either slow or minimal, as indicated by the dissimilarity in species composition between 2020–2022. The slow recovery of the community suggests that even brief episodes of MHWs can trigger persistent alterations in benthic structure, exceeding the resilience capacity of these organisms on short timescales. Determining the direct impacts caused by the high temperatures of MHWs themselves from co-occurring potential stressors (*e.g.*, changes in water nutrient content, increasing herbivory, and solar radiation) in intertidal reefs remains challenging (Straub et al., 2019), and therefore, clarifying the mechanisms behind the macroalgal beds’ recovery remains difficult, as overlapping factors may be influencing community dynamics. Despite these limitations, our study provides valuable insights into how macroalgal bed communities respond to oscillating climate conditions. Some dominant macroalgal species, highly vulnerable to MHWs, were replaced by generalist turf species after their death. The recovery of macroalgae beds may be observed after several years, as indicated by the continued shifts in the benthic assemblages.

Identifying the changes caused by MHWs and the resilience of marine ecosystems to climate change is a priority for assessing impact and informing decision-making. Such shifts will probably have significant consequences for marine biodiversity and ecosystem functioning, given that large intertidal macroalgal beds act as foundation species for numerous organisms, many of which are economically and socially important. Although simplistic, this analysis, which resembles the IPCC risk methodology, suggests that most reefs in the Eastern Marine Ecoregion of Brazil will face alternate biodiversity and trophic states as a result of MHWs. These events need to be monitored and replicated globally through LTER ecological sites, as they could provide meaningful data to evaluate the global footprints of climate-related impacts (Mieszkowska et al., 2019; Muelbert et al., 2019). Our findings, therefore, fill an important geographic gap and offer valuable ecological baselines for a rapidly warming region.

## Conclusions

Our study revealed that marine heatwaves in the SW Atlantic over the period of 2018 and 2022 were associated with significant shifts in benthic intertidal bed composition and diversity. Positive seawater temperature anomalies and periods of intense and prolonged MHWs were followed by the loss of dominant and less tolerant macroalgal species, and replacement by low diversity assemblages, supporting that intertidal coastal marine ecosystems are on a path to lower health in a warmer ocean. Our study also highlights the extreme value of long-term multiyear observations in coastal marine ecosystems and the integration of both *in situ* and remote ocean observation variables to understand the complex dynamics that marine assemblages face in the Anthropocene.

## Supplemental Information

10.7717/peerj.20858/supp-1 Supplemental Information 1 Supplementary tables with statistical results

10.7717/peerj.20858/supp-2 Supplemental Information 2 Raw data with climate and biological datasets The raw data shows meteo-oceanographic data used in analysis and benthic intertidal reef composition along the period of study. These data were used for statistical analysis to compare the effects of heatwaves in benthic assemblages.

## Additional Information and Declarations

### Field Study Permissions

The following information was supplied relating to field study approvals (i.e., approving body and any reference numbers): Field sampling was authorized by the Biodiversity Authorization and Information System of the Brazilian Institute for the Environment and Renewable Natural Resources (sampling license: SISBIO-IBAMA 57819-9).

### Data Availability

The following information was supplied regarding data availability: The raw data measurements are available in the Supplemental File.
