# Supplementary material for: Marine heatwaves shift intertidal marine communities in the SW Atlantic
Source: PeerJ. 2026 Feb 25;14:e20858. doi: 10.7717/peerj.20858 (PMC12949583; doi:10.7717/peerj.20858)
Supplement: Supplemental Information 1 [file peerj-14-20858-s001.docx]

Supplementary material for **Marine heatwaves shift intertidal marine communities in the SW Atlantic**

Mazzuco et al., Submitted to PeerJ.

Correspondence to: Angelo F. Bernardino, angelo.bernardino@ufes.br

Table S1. List of the sampling dates from December 2017 to May 2022.

|  | month | | | | | | | | | | | |
| --- | --- | --- | --- | --- | --- | --- | --- | --- | --- | --- | --- | --- |
| Year | J | F | M | A | M | J | J | A | S | O | N | D |
| 2017 |  |  |  |  |  |  |  |  |  |  |  | 18 |
| 2018 | 16 | 16 | 17 | 17 | 16 | 14 | 27 | 31 | 26 | 25 | 28 | nd |
| 2019 | 17 | 19 | 21 | 20 | 21 | 19 | 16 | 31 | 29 | 29 | 25 | 12 |
| 2020 | 12 | 12 | 11 | 7 | 22 | 25 | 22 | nd | nd | 20 | nd | nd |
| 2021 | 14 | 12 | nd | nd | 26 | nd | nd | 10 | nd | nd | 03 | 02 |
| 2022 | 18 | nd | 03 | 28 | 17 |  |  |  |  |  |  |  |

* nd: no data

Table S2. Results of analyses of variance (ANOVA) to compare the differences in average and anomalous climatic indicators between monitored years (2018, 2019, and 2020) and seasons (spring, summer, fall, winter). Note: F for statistic, significant results (p < 0.05) are in bold.

|  | **Mean** | | | | |  |  | **Anomaly** | | | | |
| --- | --- | --- | --- | --- | --- | --- | --- | --- | --- | --- | --- | --- |
| **Air T** | ***df*** | **SS** | **MS** | **F** | ***p*** |  | **Air T** | ***df*** | **SS** | **MS** | **F** | ***p*** |
| Year | 4 | 10.5 | 2.6 | 4.0 | **0.0087** |  | Year | 4 | 2.1 | 0.5 | 7.0 | **0.0003** |
| Season | 3 | 71.3 | 23.8 | 36.4 | **< 0.0001** |  | Season | 3 | 0.5 | 0.2 | 2.3 | 0.0931 |
| Year•Season | 10 | 2.6 | 0.3 | 0.4 | 0.9348 |  | Year•Season | 10 | 2.3 | 0.2 | 2.9 | **0.0081** |
| Residuals | 36 | 23.5 | 0.7 |  |  |  | Residuals | 36 | 2.7 | 0.1 |  |  |
|  |  |  |  |  |  |  |  |  |  |  |  |  |
| **Tukey HSD results:**   - 2019 ≠ 2018, 2020, 2021 - Fall ≠ Spring, Winter - Summer ≠ Spring ≠ Winter | | | | | |  | **Tukey HSD results:**   - 2019 ≠ 2018, 2020, 2021 - Year*Season (interaction): Fall 2019 ≠ Fall 2018, Fall 2020 - Year*Season (interaction): Summer 2019 ≠ Summer 2018 | | | | | |
|  |  | | | | |  |  |  |  |  |  |  |
|  | **Mean** | | | | |  |  | **Anomaly** | | | | |
| **SST** | ***df*** | **SS** | **MS** | **F** | ***p*** |  | **SST** | ***df*** | **SS** | **MS** | ***F*** | ***p*** |
| Year | 4 | 9.8 | 2.4 | 4.1 | **0.0082** |  | Year | 4 | 5.1 | 1.3 | 9.7 | **< 0.0001** |
| Season | 3 | 56.6 | 18.9 | 31.2 | **< 0.0001** |  | Season | 3 | 2.6 | 0.8 | 6.5 | **0.0012** |
| Year•Season | 10 | 2.9 | 0.3 | 0.5 | 0.8958 |  | Year•Season | 10 | 3.3 | 0.3 | 2.5 | **0.0209** |
| Residuals | 36 | 21.8 | 0.6 |  |  |  | Residuals | 36 | 4.7 | 0.1 |  |  |
|  |  |  |  |  |  |  |  |  |  |  |  |  |
| **Tukey HSD results:**   - 2019 ≠ 2021, 2022 - Fall ≠ Spring, Winter - Summer ≠ Spring, Winter | | | | | |  | **Tukey HSD results:**   - 2019 ≠ 2021, 2022 - Spring ≠ Fall - Year*Season (interaction): 2020 Fall ≠ 2020 Winter, 2020 Spring - Year*Season (interaction): 2019 Fall ≠ 2018 Fall, 2022 Fall | | | | | |
|  |  |  |  |  |  |  |  |  |  |  |  |  |
|  | **Mean** | | | | |  |  | **Anomaly** | | | | |
| **SWH** | ***df*** | **SS** | **MS** | **F** | ***p*** |  | **SWH** | ***df*** | **SS** | **MS** | **F** | ***p*** |
| Year | 4 | 1.6 | 0.03 | 1.2 | 0.3160 |  | Year | 4 | 0.0004 | 0.0001 | 0.1 | 0.9692 |
| Season | 3 | 1.4 | 0.5 | 14.6 | **< 0.0001** |  | Season | 3 | 0.02 | 0.006 | 7.3 | **0.0006** |
| Year•Season | 10 | 0.3 | 0.03 | 0.9 | 0.5320 |  | Year•Season | 10 | 0.005 | 0.0005 | 0.6 | 0.7981 |
| Residuals | 36 | 1.2 | 0.03 |  |  |  | Residuals | 36 | 0.03 | 0.001 |  |  |
|  |  |  |  |  |  |  |  |  |  |  |  |  |
| **Tukey HSD results:**  Fall ≠ Summer, Winter  Summer ≠ Spring, Winter | | | | | |  | **Tukey HSD results:**  Fall ≠ Summer, Winter  Winter ≠ Spring, Summer | | | | | |
|  |  |  |  |  |  |  |  |  |  |  |  |  |
|  | **Mean** | | | | |  |  | **Anomaly** | | | | |
| **Precipitation** | ***df*** | **SS** | **MS** | **F** | ***p*** |  | **Precipitation** | ***df*** | **SS** | **MS** | **F** | ***p*** |
| Year | 4 | 14243 | 3561 | 0.7 | 0.5904 |  | Year | 2 | 6429 | 3215 | 0.6 | 0.536 |
| Season | 3 | 88411 | 29470 | 5.8 | **0.0023** |  | Season | 3 | 9221 | 3074 | 0.6 | 0.614 |
| Year•Season | 10 | 36654 | 3665 | 0.7 | 0.6907 |  | Year•Season | 5 | 22230 | 4446 | 0.8 | 0.507 |
| Residuals | 36 | 180542 | 5015 |  |  |  | Residuals | 21 | 105161 | 5008 |  |  |
|  |  |  |  |  |  |  |  |  |  |  |  |  |
| **Tukey HSD results:**  Winter ≠ Spring, Summer | | | | | |  |  | | | | | |
|  |  |  |  |  |  |  |  |  |  |  |  |  |

Table S3. Occurrence of marine heatwaves and cold spells from December 2017 to May 2022. The longest events are highlighted in gray.

| **Event** | **Duration** | **Start date** | **Peak date** | **Maximum**  **intensity** |
| --- | --- | --- | --- | --- |
| Marine  Heatwaves | 5 | 2018-01-10 | 2018-01-13 | 1.109 |
|  | 19 | 2018-07-15 | 2018-07-25 | 1.2527 |
|  | 7 | 2018-09-27 | 2018-09-30 | 1.5895 |
|  | 10 | 2018-10-08 | 2018-10-12 | 1.5243 |
|  | 6 | 2018-11-13 | 2018-11-17 | 1.5197 |
|  | 8 | 2018-12-01 | 2018-12-06 | 1.0794 |
|  | 6 | 2019-01-10 | 2019-01-12 | 1.5046 |
|  | 5 | 2019-01-27 | 2019-01-30 | 1.675 |
|  | 7 | 2019-02-21 | 2019-02-26 | 1.5142 |
|  | 7 | 2019-03-05 | 2019-03-09 | 1.6013 |
|  | 47 | 2019-04-22 | 2019-05-09 | 4.1735 |
|  | 21 | 2019-06-14 | 2019-06-30 | 1.5617 |
|  | 5 | 2019-10-10 | 2019-10-12 | 1.2443 |
|  | 29 | 2019-10-27 | 2019-11-12 | 1.8541 |
|  | 5 | 2019-12-19 | 2019-12-21 | 1.4355 |
|  | 5 | 2020-06-25 | 2020-06-28 | 1.0741 |
|  | 31 | 2020-07-23 | 2020-08-13 | 1.4277 |
|  | 12 | 2020-09-14 | 2020-09-16 | 1.3305 |
|  | 14 | 2020-09-29 | 2020-10-02 | 1.9652 |
|  | 5 | 2020-11-17 | 2020-11-19 | 1.1084 |
|  | 11 | 2020-11-29 | 2020-11-30 | 1.0669 |
|  | 12 | 2021-09-12 | 2021-09-18 | 1.4465 |
|  |  |  |  |  |
| Marine  cold spells | 7 | 2018-02-06 | 2018-02-10 | -1.4107 |
|  | 7 | 2018-02-27 | 2018-03-05 | -1.6818 |
|  | 5 | 2020-04-10 | 2020-04-14 | -1.4603 |
|  | 26 | 2020-04-26 | 2020-05-12 | -2.3863 |
|  | 7 | 2020-05-26 | 2020-05-29 | -1.4977 |

Table S4. Results of 1-way analyses of variance (ANOVA) to compare the differences in low-tide heatwaves and high-wave rainstorms between seasons and years. Note: F for statistic, significant results (p < 0.05) are in bold.

|  | **Low-tide heatwaves** | | | | |  |  | **High-wave rainstorm** | | | | |
| --- | --- | --- | --- | --- | --- | --- | --- | --- | --- | --- | --- | --- |
| **Duration** | ***df*** | **SS** | **MS** | **F** | ***p*** |  | **Duration** | ***df*** | **SS** | **MS** | **F** | ***p*** |
| Year | 5 | 561.5 | 112.3 | 5.1 | **0.0013** |  | Year | 5 | 0.8 | 0.2 | 0.6 | 0.6514 |
| Season | 3 | 602.8 | 200.9 | 9.1 | **0.0001** |  | Season | 3 | 1.8 | 0.6 | 2.5 | 0.0759 |
| Year•Season | 10 | 662.0 | 66.2 | 3.0 | **0.0077** |  | Year•Season | 10 | 4.1 | 0.4 | 1.7 | 0.1196 |
| Residuals | 35 | 771.2 | 22.0 |  |  |  | Residuals | 35 | 8.5 | 0.2 |  |  |
| **Tukey HSD results:**  2019 ≠ 2021, 2022  Summer ≠ Spring, Winter ≠ Fall  Year*Season (interaction): Fall 2019 ≠ 2020, 2021, 2022 Fall | | | | | |  |  | | | | | |
|  |  | | | | |  |  |  |  |  |  |  |
|  | **Low-tide heatwaves** | | | | |  |  | **High-wave rainstorm** | | | | |
| **Number of events** | ***df*** | **SS** | **MS** | **F** | ***p*** |  | **Number of events** | ***df*** | **SS** | **MS** | ***F*** | ***p*** |
| Year | 5 | 3.7 | 0.7 | 1.2 | 0.3344 |  | Year | 5 | 0.8 | 0.2 | 0.8 | 0.5699 |
| Season | 3 | 13.9 | 4.6 | 7.4 | **0.0005** |  | Season | 3 | 1.6 | 0.5 | 2.6 | 0.0686 |
| Year•Season | 10 | 4.2 | 0.4 | 0.6 | 0.7491 |  | Year•Season | 10 | 3.3 | 0.3 | 1.6 | 0.1472 |
| Residuals | 35 | 22.0 | 0.6 |  |  |  | Residuals | 35 | 7.2 | 0.2 |  |  |
| **Tukey HSD results:**  Spring ≠ Fall, Summer | | | | | |  |  | | | | | |
|  |  |  |  |  |  |  |  |  |  |  |  |  |
|  | **Low-tide heatwaves** | | | | |  |  | **High-wave rainstorm** | | | | |
| **Low-tide height** | ***df*** | **SS** | **MS** | **F** | ***p*** |  | **SWH** | ***df*** | **SS** | **MS** | **F** | ***p*** |
| Year | 5 | 0.1 | 0.02 | 2.0 | 0.096 |  | Year | 5 | 3.4 | 0.7 | 1.5 | 0.1988 |
| Season | 3 | 0.01 | 0.003 | 0.3 | 0.802 |  | Season | 3 | 6.3 | 2.1 | 4.8 | **0.0063** |
| Year•Season | 10 | 0.1 | 0.01 | 0.8 | 0.541 |  | Year•Season | 10 | 8.2 | 0.8 | 1.9 | 0.0826 |
| Residuals | 35 | 0.4 | 0.01 |  |  |  | Residuals | 35 | 15.3 | 0.4 |  |  |
|  |  |  |  |  |  |  | **Tukey HSD results:**  Fall ≠ Summer, Winter | | | | | |
|  |  |  |  |  |  |  |  |  |  |  |  |  |
|  | **Low-tide heatwaves** | | | | |  |  | **High-wave rainstorm** | | | | |
| **SST** | ***df*** | **SS** | **MS** | **F** | ***p*** |  | **Precipitation** | ***df*** | **SS** | **MS** | **F** | ***p*** |
| Year | 5 | 1.5 | 0.3 | 3.2 | **0.0173** |  | Year | 5 | 1470 | 294.0 | 0.9 | 0.4620 |
| Season | 3 | 0.9 | 0.3 | 2.9 | **0.0456** |  | Season | 3 | 2581 | 860.2 | 2.7 | 0.0556 |
| Year•Season | 10 | 1.9 | 0.2 | 2.0 | 0.0603 |  | Year•Season | 10 | 3228 | 322.8 | 1.0 | 0.4305 |
| Residuals | 35 | 3.4 | 0.1 |  |  |  | Residuals | 35 | 10843 | 309.8 |  |  |
| **Tukey HSD results:**  2021 ≠ 2019, 2022  Winter ≠ Summer | | | | | |  |  | | | | | |
|  |  |  |  |  |  |  |  |  |  |  |  |  |

Table S5. List of high taxonomic composition (HTC) registered in the magroalgal beds.

|  | **HTC** | **Aphia** |
| --- | --- | --- |
| Anemonae | *Bunodosoma* | urn:lsid:marinespecies.org:taxname:100700 |
| Anemonae | *Actinostella* | urn:lsid:marinespecies.org:taxname:267202 |
| Anthozoa | *Palythoa* | urn:lsid:marinespecies.org:taxname:205785 |
| Anthozoa | *Siderastrea* | urn:lsid:marinespecies.org:taxname:204291 |
| Anthozoa | *Zoanthus* | urn:lsid:marinespecies.org:taxname:206284 |
| Bivalvia | *Mytilaster* | urn:lsid:marinespecies.org:taxname:138227 |
| Crustacea | *Chthamalus* | urn:lsid:marinespecies.org:taxname:106126 |
| Crustacea | Anomura | urn:lsid:marinespecies.org:taxname:106671 |
| Echinodermata | *Echinaster* | urn:lsid:marinespecies.org:taxname:123275 |
| Echinodermata | *Echinometra* | urn:lsid:marinespecies.org:taxname:179659 |
| Gastropoda | Gastropoda | urn:lsid:marinespecies.org:taxname:101 |
| Gastropoda | *Tegula* | urn:lsid:marinespecies.org:taxname:413467 |
| Gastropoda | *Aplysia* | urn:lsid:marinespecies.org:taxname:137654 |
| Gastropoda | *Bursatella* | urn:lsid:marinespecies.org:taxname:137655 |
| Porifera | Porifera | urn:lsid:marinespecies.org:taxname:558 |
| Rhodophyta | *Amphiroa* | urn:lsid:marinespecies.org:taxname:144003 |
| Rhodophyta | *Asparagopsis* | urn:lsid:marinespecies.org:taxname:143809 |
| Rhodophyta | *Botryocladia* | urn:lsid:marinespecies.org:taxname:144247 |
| Rhodophyta | *Corallina* | urn:lsid:marinespecies.org:taxname:144007 |
| Rhodophyta | *Cryptonemia* | urn:lsid:marinespecies.org:taxname:144042 |
| Rhodophyta | *Dichotomaria* | urn:lsid:marinespecies.org:taxname:369570 |
| Rhodophyta | *Gelidium* | urn:lsid:marinespecies.org:taxname:144135 |
| Rhodophyta | *Gracilaria* | urn:lsid:marinespecies.org:taxname:144188 |
| Rhodophyta | *Gymnogongrus* | urn:lsid:marinespecies.org:taxname:144168 |
| Rhodophyta | *Hypnea* | urn:lsid:marinespecies.org:taxname:144157 |
| Rhodophyta | *Jania* | urn:lsid:marinespecies.org:taxname:144012 |
| Rhodophyta | *Liagora* | urn:lsid:marinespecies.org:taxname:144211 |
| Rhodophyta | *Lithophyllum* | urn:lsid:marinespecies.org:taxname:144016 |
| Rhodophyta | *Ochtodes* | urn:lsid:marinespecies.org:taxname:369959 |
| Rhodophyta | *Plocamium* | urn:lsid:marinespecies.org:taxname:144223 |
| Rhodophyta | *Pterocladiella* | urn:lsid:marinespecies.org:taxname:144137 |
| Rhodophyta | *Spyridia* | urn:lsid:marinespecies.org:taxname:143866 |
| Rhodophyta | *Osmundaria* | urn:lsid:marinespecies.org:taxname:143920 |
| Chlorophyta | *Anadyomene* | urn:lsid:marinespecies.org:taxname:143992 |
| Chlorophyta | *Caulerpa* | urn:lsid:marinespecies.org:taxname:143816 |
| Chlorophyta | *Willeella* | urn:lsid:marinespecies.org:taxname:206638 |
| Chlorophyta | *Cladophora* | urn:lsid:marinespecies.org:taxname:143996 |
| Chlorophyta | *Dictyosphaeria* | urn:lsid:marinespecies.org:taxname:144265 |
| Chlorophyta | *Halimeda* | urn:lsid:marinespecies.org:taxname:143820 |
| Chlorophyta | *Ulva* | urn:lsid:marinespecies.org:taxname:144296 |
| Phaeophyta | *Canistrocarpus* | urn:lsid:marinespecies.org:taxname:369437 |
| Phaeophyta | *Colpomenia* | urn:lsid:marinespecies.org:taxname:144253 |
| Phaeophyta | *Dictyopteris* | urn:lsid:marinespecies.org:taxname:144085 |
| Phaeophyta | *Dictyota* | urn:lsid:marinespecies.org:taxname:144086 |
| Phaeophyta | *Ectocarpales* | urn:lsid:marinespecies.org:taxname:842 |
| Phaeophyta | *Hydroclathrus* | urn:lsid:marinespecies.org:taxname:144255 |
| Phaeophyta | *Lobophora* | urn:lsid:marinespecies.org:taxname:144087 |
| Phaeophyta | *Padina* | urn:lsid:marinespecies.org:taxname:144088 |
| Phaeophyta | *Sargassum* | urn:lsid:marinespecies.org:taxname:144132 |
| Phaeophyta | *Spatoglossum* | urn:lsid:marinespecies.org:taxname:144090 |
| Phaeophyta | *Stypopodium* | urn:lsid:marinespecies.org:taxname:144091 |
| Phaeophyta | *Zonaria* | urn:lsid:marinespecies.org:taxname:144093 |

Table S6. Results of 3-way analyses of variance (ANOVA) to compare the differences in benthic % cover (macroalgae, corals, and other epibenthos), diversity (Shannon-Wiener Index S-W), and richness (number of species/taxa) between months, seasons, and years. Note: F for statistic, significant results (*p* < 0.05) are in bold. Data was Log(x+10) or square-root transformed prior to the analysis.

|  | **Macroalgae Cover** | | | | |  | **Diversity** | | | | |
| --- | --- | --- | --- | --- | --- | --- | --- | --- | --- | --- | --- |
|  | ***df*** | **SS** | **MS** | **F** | ***p*** |  | ***df*** | **SS** | **MS** | **F** | ***p*** |
| Year | 4 | 20.9 | 5.2 | 25.7 | **< 0.0001** |  | 4 | 6.1 | 1.5 | 21.1 | **< 0.0001** |
| Season | 3 | 1.9 | 0.7 | 3.2 | **0.0250** |  | 3 | 1.2 | 0.4 | 5.7 | **0.0009** |
| (Month)Season | 8 | 3.7 | 0.5 | 2.3 | **0.0254** |  | 8 | 1.8 | 0.2 | 3.0 | **0.0029** |
| Y*S | 10 | 3.1 | 0.3 | 1.5 | 0.1318 |  | 10 | 2.6 | 0.3 | 3.6 | **0.0002** |
| Y*M | 16 | 12.9 | 0.8 | 3.9 | **< 0.0001** |  | 16 | 3.5 | 0.2 | 3.0 | **0.0001** |
| Residuals | 168 | 34.1 | 0.2 |  |  |  | 168 | 12.0 | 0.1 |  |  |
|  |  |  |  |  |  |  |  |  |  |  |  |
|  | **Coral Cover** | | | | |  | **Richness** | | | | |
|  | ***df*** | **SS** | **MS** | **F** | ***p*** |  | ***df*** | **SS** | **MS** | **F** | ***p*** |
| Year | 4 | 6.3 | 1.6 | 5.9 | **0.0002** |  | 4 | 104.2 | 26.1 | 7.6 | **< 0.0001** |
| Season | 3 | 1.7 | 0.5 | 2.1 | 0.1050 |  | 3 | 77.7 | 25.9 | 7.6 | **0.0001** |
| (Month)Season | 8 | 4.2 | 0.5 | 1.9 | 0.0516 |  | 8 | 90.4 | 11.3 | 3.3 | **0.0043** |
| Y*S | 10 | 5.8 | 0.6 | 2.2 | **0.0195** |  | 10 | 156.9 | 15.7 | 4.6 | 0.2833 |
| Y*M | 16 | 7.9 | 0.5 | 1.9 | **0.0261** |  | 16 | 140.9 | 8.8 | 2.6 | **0.0391** |
| Residuals | 168 | 44.3 | 0.3 |  |  |  | 168 | 575.3 | 3.4 |  |  |
|  |  |  |  |  |  |  |  |  |  |  |  |
|  | **Others Cover** | | | | |  |  |  |  |  |  |
|  | ***df*** | **SS** | **MS** | **F** | ***p*** |  |  |  |  |  |  |
| Year | 4 | 45.7 | 11.4 | 88.9 | **< 0.0001** |  |  |  |  |  |  |
| Season | 3 | 0.3 | 0.1 | 0.9 | 0.4377 |  |  |  |  |  |  |
| (Month)Season | 8 | 4.3 | 0.5 | 4.2 | **0.0001** |  |  |  |  |  |  |
| Y*S | 10 | 11.3 | 1.1 | 8.8 | **< 0.0001** |  |  |  |  |  |  |
| Y*M | 16 | 7.5 | 0.5 | 3.7 | **< 0.0001** |  |  |  |  |  |  |
| Residuals | 168 | 21.6 | 1.1 |  |  |  |  |  |  |  |  |
|  |  |  |  |  |  |  |  |  |  |  |  |

Table S7. Significant Tukey HSD results (p < 0.05) to assess pairwise differences in total % cover (Macroalgae, Coral, Other epibenthos), diversity, and richness.

| **Tukey HSD results** | | |
| --- | --- | --- |
| **Macroalgae** |  |  |
| **Year:**   - 2018 ≠ 2019, 2021, 2022 - 2019 ≠ 2020, 2021, 2022 - 2022 ≠ 2020, 2021 | **Year*Month:**   - 2018Jun ≠ 2018Aug, 2019(Apr, Jul, Nov) 2020Mar - 2021Aug ≠ 2017Dec, 2018(Aug, Jan, May, Nov, Oct), 2019(Apr, Dec, Feb, Jan, Jul, Jun, Mar, May, Nov, Oct, Sep), 2020(Jan, Jun, Mar) - 2022Jan ≠ 2018(Aug, Jan, May, Nov), 2019(Apr, Aug, Dec, Feb, Jan, Jul, Nov, Oct, Sep), 2020(Jan, Mar) | **Year*Month:**   - 2022Mar ≠ 2018Aug, 2019(Apr, Nov) 2020Mar - 2022May ≠ 2017Dec, 2018(Apr, Aug, Feb, Jan, Jul, Mar, May, Nov, Oct, Sep), 2019(all months), 2020(Apr, Feb, Jan, Jul, Jun, Mar, May, Oct), 2021(Dec, Feb, Jan, May, Nov), 2022Apr |
| **Coral** |  |  |
| **Year:**   - 2022 ≠ 2018, 2019, 2020 | **Year*Month:**   - 2020Jun ≠ 2019Oct, 2021(Jan, Aug), 2020Oct, 2022(Mar, May) |  |
| **Other epibenthos** |  |  |
| **Year:**   - 2018 ≠ 2019, 2021 - 2019 ≠ 2020, 2021, 2022 - 2020 ≠ 2021, 2022 | **Year*Month:**   - 2018Jun ≠ 2018(Nov, Oct), 2019(Apr, Jun, May), 2020Jan - 2018Sep ≠ 2018Oct, 2019(Apr, Jun, May), 2020Jan - 2019Jan ≠ 2017Dec, 2018(Jan, Feb, Mar, Apr, May, Jun, Jul, Aug, Sep, Nov), 2019(Dec, Jun, Mar), 2020(Feb, Mar, May, Jun, Jul), 2021Aug, 2022(Apr, Mar) - 2019Feb ≠ 2017Dec, 2018(Apr, Aug, Jan, Mar, Jun, Jul, May, Sep), 2020(Mar, Apr, Jun), 2021Aug, 2022Mar - 2019Jul ≠ 2017Dec, 2018(Apr, Jan, Mar, Jun, Jul, May, Sep), 2020(Mar, Apr), 2021Aug, 2022Mar - 2019Aug ≠ 2017Dec, 2018(Apr, Aug, Jan, Mar, Jun, Jul, May, Sep), 2020(Jun, Mar, Apr, May), 2021Aug, 2022(Apr, Mar) - 2019Sep ≠ 2017Dec, 2018(Apr, Aug, Jan, Feb, Mar, Jun, Jul, May, Sep), 2020(Feb, Jun, Mar, Apr, May), 2021Aug, 2022(Apr, Mar) | **Year•Month:**   - 2019Oct ≠ 2017Dec, 2018(Apr, Aug, Jan, Feb, Mar, Jun, Jul, May, Sep), 2019(Dec, Mar, Feb, Jul, Jun, Mar, Apr, May), 2021Aug, 2022(Apr, Mar) - 2019Nov ≠ 2017Dec, 2018(Apr, Aug, Jan, Mar, Jun, Jul, May, Sep), 2020(Mar, Apr) - 2020Oct ≠ 2018(Nov, Oct), 2019(Apr, Aug, Feb, Jan, Jul, Jun, Mar, May, Nov, Oct, Sep), 2020Jan, 2022Mar - 2021Jan ≠ 2018(Nov, Oct), 2019(all months), 2020Jan, 2020Jul - 2021Feb ≠ 2018(Nov, Oct), 2019(Apr, Aug, Feb, Jan, Jul, Jun, Mar, May, Nov, Oct, Sep), 2020Jan - 2021May ≠ 2018Oct, 2019(all months), 2020Jan - 2021Nov ≠ 2018(Feb, Nov, 2018Oct, 2019(all months), 2020(Feb, Jan, Jul) - 2021Dec and 2022Jan ≠ 2018(Nov, Oct), 2019(all months), 2020(Jan, Jul) - 2022May ≠ 2018(Feb, Nov, Oct), 2019(all months), 2020(Feb, Jan, Jul) |
| **Diversity** |  |  |
| **Year:**   - 2019 ≠ 2018, 2020, 2021, 2022   **Season:**   - Spring ≠ Fall, Winter   **Month:**   - Nov ≠ Apr, May, Jun | **Year•Month:**   - 2018Apr ≠ 2018(Feb, Nov, Oct), 2019(Jan, Feb, Apr, May, Jul, Aug, Sep, Oct, Nov, Dec), 2020(Jan, Mar), 2021Nov - 2018Sep ≠ 2018Jan, 2019(Feb, Apr, Aug, Sep), 2021Nov - 2019Jan ≠ 2018(Jan, Mar, May, Sep), 2019Jun, 2020(Apr, May, Jul, Oct), 2021(Dec, Feb), 2022Apr - 2019Oct and 2019Nov ≠ 2018(Jan, Sep), 2020(Jul, May) | **Year•Month:**   - 2020Feb ≠ 2019(Jan, Apr, Sep, Oct, Nov), 2021Nov - 2020Jun ≠ 2018Oct, 2019(Jan, Feb, Apr, Jul, Aug, Sep, Oct, Nov), 2020Mar, 2021Nov - 2021Aug ≠ 2019(Jan, Feb, Apr, Aug, Sep, Oct, Nov), 2020Mar, 2021Nov - 2021Nov ≠ 2018Jan |
| **Richness** |  |  |
| **Year:**   - 2019 ≠ 2018, 2020   **Season:**   - Winter ≠ Spring, Summer   **Month:**   - Nov ≠ Apr, Jun, Jul, Aug, Sep | **Year•Month:**   - 2018Apr ≠ 2018(Feb, Aug, Oct, Nov), 2019(Jan, Feb, Mar, Apr, May, Jul, Aug, Oct, Nov, Dec), 2020(Jan, Mar), 2021(Jan, May, Nov, Dec), 2022(Jan, Mar) - 2019Jan ≠ 2018Jan, 2020(Jul, Jun), 2021Aug | **Year•Month:**   - 2019Nov ≠ 2018Jan, 2020Jul, 2020Jun, 2022Apr - 2021Aug ≠ 2018Oct, 2019(Apr, Dec, Feb, Jan, Nov), 2020Mar, 2021(Jan, May) - 2021Nov ≠ 2018(Jan, Sep), 2020(Feb, Jul, Jun), 2021Aug, 2022Apr |

Table S8. Results of 1-way ANOVA to compare the differences in benthic % cover (macroalgae, corals, and other epibenthos) obtained by visual count between years across the region (LTER site 1, site 2, site 3). Note: F for statistic, significant results (*p* < 0.05) are in bold; data was log(x+1) transformed.

|  | **Site 1 (Gramute)** | | | | |  | **Site 2 (Enseada das Garças)** | | | | |  | **Site 3 (Costa Bela)** | | | | |
| --- | --- | --- | --- | --- | --- | --- | --- | --- | --- | --- | --- | --- | --- | --- | --- | --- | --- |
|  | **Macroalgae** | | | | |  | **Macroalgae** | | | | |  | **Macroalgae** | | | | |
|  | ***df*** | **SS** | **MS** | **F** | ***p*** |  | ***df*** | **SS** | **MS** | **F** | ***p*** |  | ***df*** | **SS** | **MS** | **F** | ***p*** |
| Year | 2 | 2.3 | 1.2 | 10.8 | **0.0102** |  | 2 | 19.6 | 9.8 | 33.9 | 0.0005 |  | 2 | 0.9 | 0.4 | 0.6 | 0.5695 |
| Residuals | 6 | 0.6 | 0.1 |  |  |  | 6 | 1.7 | 0.3 |  |  |  | 6 | 4.4 | 0.7 |  |  |
|  |  |  |  |  |  |  |  |  |  |  |  |  |  |  |  |  |  |
| Tukey HSD result: 2018 ≠ 2019 and 2020 | | | | | |  | 2019 ≠ 2018, 2020 | | | | |  |  | | | | |
|  |  | | | | |  |  |  |  |  |  |  |  |  |  |  |  |
|  | **Corals** | | | | |  | **Corals** | | | | |  | **Corals** | | | | |
|  | ***df*** | **SS** | **MS** | **F** | ***p*** |  | ***df*** | **SS** | **MS** | **F** | ***p*** |  | ***df*** | **SS** | **MS** | **F** | ***p*** |
| Year | 2 | 5.4 | 2.7 | 7.9 | **0.0208** |  | 2 | 0.7 | 0.3 | 1.7 | 0.2693 |  | 2 | 1.6 | 0.8 | 3.2 | 0.1103 |
| Residuals | 6 | 2.0 | 0.3 |  |  |  | 6 | 1.3 | 0.2 |  |  |  | 6 | 1.5 | 0.2 |  |  |
|  |  |  |  |  |  |  |  |  |  |  |  |  |  |  |  |  |  |
| Tukey HSD result: 2018 ≠ 2019 and 2020 | | | | | |  |  |  |  |  |  |  |  |  |  |  |  |
|  |  | | | | |  |  | | | | |  |  | | | | |
|  | **Other** | | | | |  | **Other** | | | | |  | **Other** | | | | |
|  | ***df*** | **SS** | **MS** | **F** | ***p*** |  | ***df*** | **SS** | **MS** | **F** | ***p*** |  | ***df*** | **SS** | **MS** | **F** | ***p*** |
| Year | 2 | 24.4 | 12.2 | 482.2 | **< 0.0001** |  | 2 | 25.1 | 12.5 | 3059.6 | **< 0.0001** |  | 2 | 2.2 | 1.1 | 2.6 | 0.1505 |
| Residuals | 6 | 0.2 | 0.02 |  |  |  | 6 | 0.02 | 0.0 |  |  |  | 6 | 2.5 | 0.4 |  |  |
|  |  |  |  |  |  |  |  |  |  |  |  |  |  |  |  |  |  |
| Tukey HSD result: 2018 ≠ 2019 ≠ 2020 |  |  |  |  |  |  | 2018 ≠ 2019 ≠ 2020 | | | | |  |  |  |  |  |  |
|  |  |  |  |  |  |  |  |  |  |  |  |  |  |  |  |  |  |

Table S9. PERMANOVA results to compare inter-annual macroalgal beds’ % cover across the region (LTER site 1, site 2, site 3). Note: F for statistic, significant results (*p* < 0.05) are in bold; data was square-root transformed.

|  | **Site 1 (Gramute)** | | | | |  | **Site 2 (Enseada das Garças)** | | | | |  | **Site 3 (Costa Bela)** | | | | |
| --- | --- | --- | --- | --- | --- | --- | --- | --- | --- | --- | --- | --- | --- | --- | --- | --- | --- |
|  | **Rhodophyta** | | | | |  | **Rhodophyta** | | | | |  | **Rhodophyta** | | | | |
|  | ***df*** | **SS** | **MS** | **F** | ***p*** |  | ***df*** | **SS** | **MS** | **F** | ***p*** |  | ***df*** | **SS** | **MS** | **F** | ***p*** |
| Year | 1 | 0.6 | 0.6 | 5.2 | **0.02** |  | 1 | 0.1 | 0.1 | 10.2 | **0.01** |  | 1 | 0.2 | 0.2 | 2.5 | 0.12 |
| Residuals | 28 | 3.3 | 0.1 |  |  |  | 28 | 0.3 | 0.01 |  |  |  | 28 | 2.6 | 0.1 |  |  |
| Total | 29 | 3.9 |  |  |  |  | 29 | 0.4 |  |  |  |  | 29 | 2.9 |  |  |  |
| Tukey HSD result: 2018 ≠ 2019, 2020 | | | | | |  | 2018 ≠ 2019 ≠ 2020 | | | | |  |  | | | | |
|  |  | | | | |  |  |  |  |  |  |  |  |  |  |  |  |
|  | **Phaeophyta** | | | | |  | **Phaeophyta** | | | | |  | **Phaeophyta** | | | | |
|  | ***df*** | **SS** | **MS** | **F** | ***p*** |  | ***df*** | **SS** | **MS** | **F** | ***p*** |  | ***df*** | **SS** | **MS** | **F** | ***p*** |
| Year | 1 | 0.4 | 0.4 | 22.7 | **0.01** |  | 1 | 0.6 | 0.6 | 41.0 | **0.01** |  | 1 | 0.1 | 0.1 | 3.6 | **0.03** |
| Residuals | 28 | 0.5 | 0.02 |  |  |  | 28 | 0.4 | 0.01 |  |  |  | 28 | 0.4 | 0.02 |  |  |
| Total | 29 | 0.9 |  |  |  |  | 29 | 1.0 |  |  |  |  | 29 | 0.5 |  |  |  |
| Tukey HSD result: 2018 ≠ 2019, 2020 | | | | | |  |  |  | 2018 ≠ 2019 ≠ 2020 |  |  |  | 2018 ≠ 2020 | | | | |
|  |  | | | | |  |  | | | | |  |  | | | | |
|  | **Chlorophyta** | | | | |  | **Chlorophyta** | | | | |  | **Chlorophyta** | | | | |
|  | ***df*** | **SS** | **MS** | **F** | ***p*** |  | ***df*** | **SS** | **MS** | **F** | ***p*** |  | ***df*** | **SS** | **MS** | **F** | ***p*** |
| Year | 1 | 0.02 | 0.02 | 5.9 | **0.01** |  | 1 | 11.9 | 0.004 | 3.9 | **0.03** |  | 1 | 0.0004 | 0.0004 | 0.9 | 0.4 |
| Residuals | 28 | 0.07 | 0.002 |  |  |  | 28 | 6.4 | 0.001 |  |  |  | 28 | 0.01 | 0.0005 |  |  |
| Total | 29 | 0.09 |  |  |  |  | 29 | 1.2 |  |  |  |  | 29 | 0.01 |  |  |  |
| Tukey HSD result: 2018 ≠ 2019 ≠ 2020 | | | | | |  | 2018 ≠ 2019, 2020 | | | | |  |  | | | | |
|  |  | | | | |  |  | | | | |  |  | | | | |
|  | **Corals** | | | | |  | **Corals** | | | | |  | **Corals** | | | | |
|  | ***df*** | **SS** | **MS** | **F** | ***p*** |  | ***df*** | **SS** | **MS** | **F** | ***p*** |  | ***df*** | **SS** | **MS** | **F** | ***p*** |
| Year | 1 | 0.07 | 0.07 | 14.6 | **0.01** |  | 1 | 0.03 | 0.04 | 2.0 | 0.11 |  | 1 | 0.2 | 0.2 | 24.5 | **0.01** |
| Residuals | 28 | 0.1 | 0.005 |  |  |  | 28 | 0.5 | 0.02 |  |  |  | 28 | 0.2 | 0.007 |  |  |
| Total | 29 | 0.2 |  |  |  |  | 29 | 0.5 |  |  |  |  | 29 | 0.4 |  |  |  |
| Tukey HSD result: 2018 ≠ 2019, 2020 | | | | | |  |  | | | | |  | 2018 ≠ 2019 ≠ 2020 | | | | |
|  | | | | | |  |  | | | | |  |  | | | | |
|  | **Other** | | | | |  | **Other** | | | | |  | **Other** | | | | |
|  | ***df*** | **SS** | **MS** | **F** | ***p*** |  | ***df*** | **SS** | **MS** | **F** | ***p*** |  | ***df*** | **SS** | **MS** | **F** | ***p*** |
| Year | 1 | 0.2 | 0.2 | 30.7 | **0.01** |  | 1 | 0.2 | 0.2 | 87.5 | **0.01** |  | 1 | 0.1 | 0.1 | 39.4 | **0.01** |
| Residuals | 28 | 0.1 | 0.06 |  |  |  | 28 | 0.1 | 0.02 |  |  |  | 28 | 0.1 | 0.02 |  |  |
| Total | 29 | 0.3 |  |  |  |  | 29 | 0.3 |  |  |  |  | 29 | 0.2 |  |  |  |
| Tukey HSD result: 2018 ≠ 2019 ≠ 2020 | | | | | |  | 2018 ≠ 2019 ≠ 2020 | | | | |  | 2020 ≠ 2018, 2019 | | | | |
|  |  |  |  |  |  |  |  |  |  |  |  |  |  |  |  |  |  |

Table S10. CAP results for the ordination of benthic cover with MHWs Events, duration, SST and SSH

| Importance of components | CAP1 | CAP2 |  |  |
| --- | --- | --- | --- | --- |
| Eigenvalue | 0.586 | 0.332 |  |  |
| Proportion explained | 0.540 | 0.306 |  |  |
| Cumulative proportion | 0.540 | 0.846 |  |  |
|  | CAP1 | CAP2 | F | p |
| Events | 0.682 | 0.181 | 1.950 | **0.056** |
| Duration | 0.797 | -0.314 | 1.606 | 0.122 |
| SST | 0.353 | -0.325 | 1.603 | 0.105 |
| SSH | -0.054 | -0.570 | 1.460 | 0.158 |
|  | df | SS | F | p |
| Model | 4 | 1.086 | 1.654 | **0.027** |
| Residual | 37 | 6.071 |  |  |


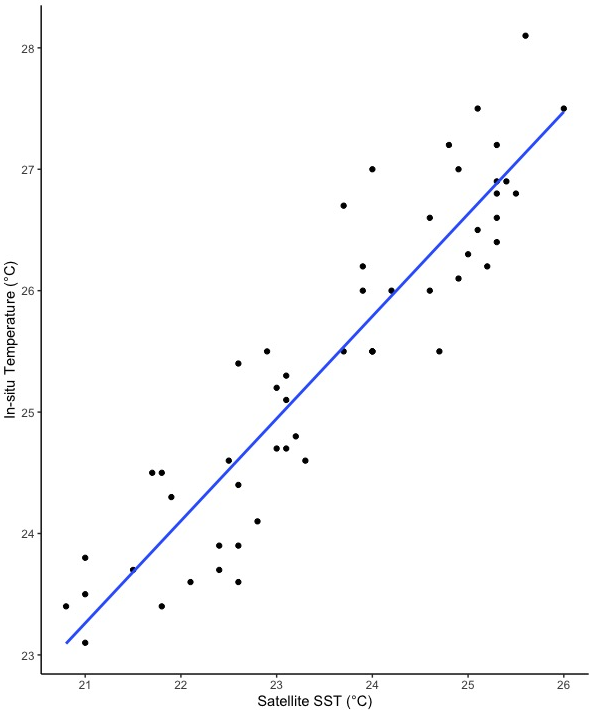


Figure S1. Spearman correlation between satellite SST and in situ temperature logger at the studied area t = 16.605 df = 52 R= 0.917 p < 0.001


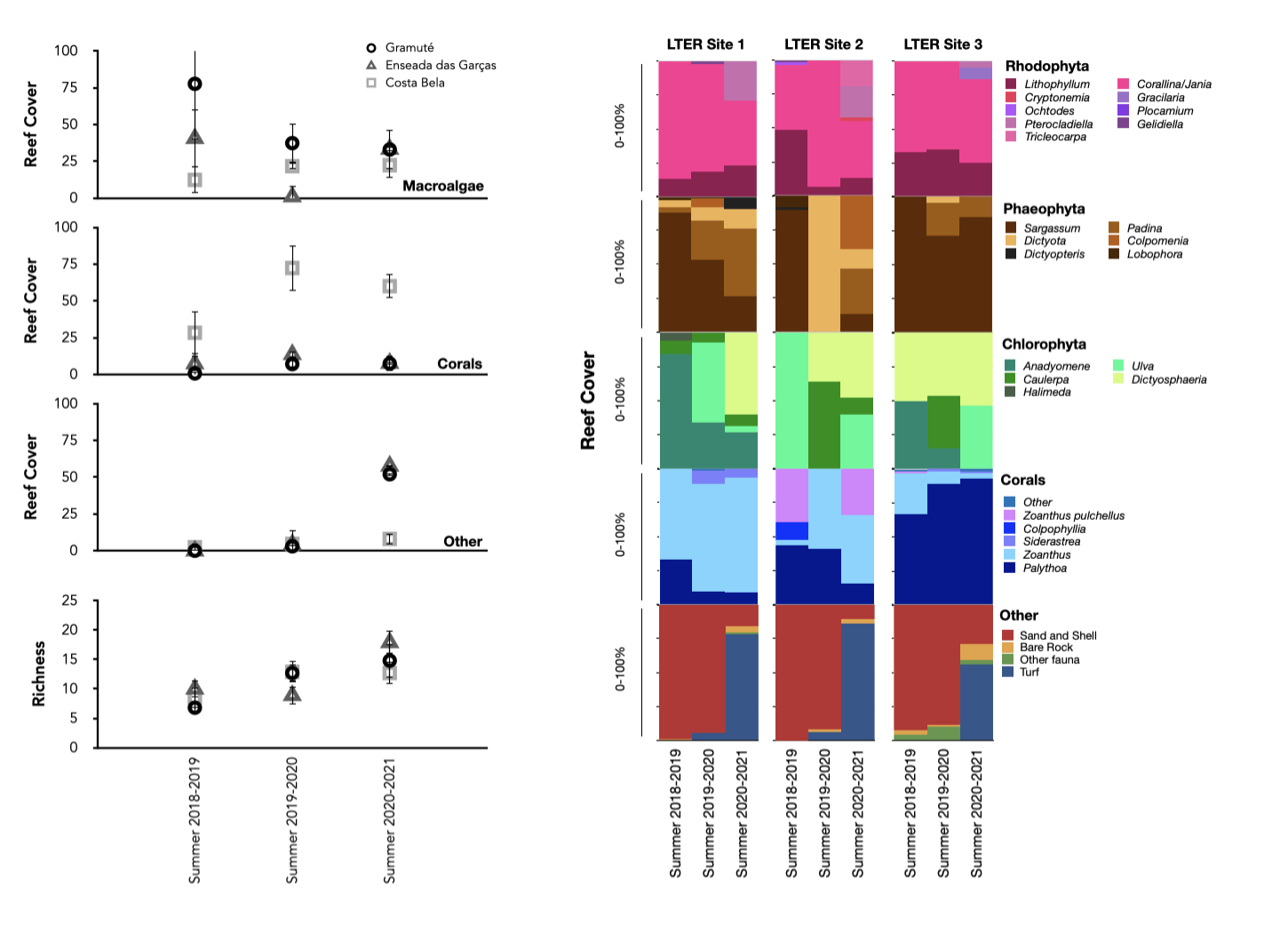


Figure S2. Differences in benthic cover (total and relative) and richness in the macroalgal beds across the study region.
